# Systemic Inflammatory Burden Causes Liver Injury in H1N1-Infected Mice

**DOI:** 10.3390/v17081132

**Published:** 2025-08-18

**Authors:** Junbin Wang, Qing Huang, Yun Yang, Cong Tang, Wenhai Yu, Yanan Zhou, Daoju Wu, Bai Li, Hao Yang, Haixuan Wang, Lei Ma, Shuaiyao Lu

**Affiliations:** 1 Institute of Medical Biology, Chinese Academy of Medical Sciences and Peking Union Medical College, Kunming 650031, China; wjb@imbcams.com.cn (J.W.); qinghuangfgh@163.com (Q.H.); yangyun.imbcams@outlook.com (Y.Y.); tangcong20210317@163.com (C.T.); wenhaiyu1234@163.com (W.Y.); 15887034059@163.com (Y.Z.); wudaoju@imbcams.com.cn (D.W.); libai119236714@126.com (B.L.); 15911606461@163.com (H.Y.); wanghaixuan@imbcams.com.cn (H.W.); 2 State Key Laboratory of Respiratory Health and Multimorbidity, Beijing 100005, China; 3 Key Laboratory of Pathogen Infection Prevention and Control (Peking Union Medical College), Ministry of Education, Beijing 102629, China; 4 Yunnan Key Laboratory of Cross-Border Infectious Disease Control and Prevention and Novel Drug Development, Kunming 650031, China; 5 Yunnan Provincial Key Laboratory of Vector-Borne Diseases Control and Research, Kunming 650031, China

**Keywords:** H1N1, liver injury, cytokine, systemic inflammation

## Abstract

Clinical evidence has associated H1N1 influenza with liver impairment, yet the underlying mechanisms remain poorly understood. Here, we investigated H1N1-induced liver damage and its potential mechanisms using a BALB/c mouse infection model. Pathological examination and serum aspartate transaminase (AST) and alanine transaminase (ALT) were assessed. Messenger ribonucleic acid-sequence was used to analyze the transcriptomic changes in tissues. Multiple inflammatory cytokines in tissues and inflammatory cells in the blood were detected on the fifth day post-infection. Our results showed that H1N1 infection caused significant liver pathology and elevated serum AST/ALT levels. Transcriptomic analysis revealed significant alterations in liver gene expression profiles following H1N1 infection, particularly in genes associated with inflammatory responses, including those involved in monocyte adhesion/activation and neutrophil/macrophage infiltration. Marked increases in inflammatory mediators were observed in lungs, serum, and liver, accompanied by systemic changes in circulating inflammatory cells, indicating H1N1 triggered a robust systemic inflammatory response. These findings suggest that H1N1-induced liver damage may be associated with the systemic inflammatory response induced by H1N1 and changes in liver gene regulation.

## 1. Introduction

In spring 2009, a novel strain of influenza A virus, known as the pandemic A(H1N1)pdm09 virus, first emerged in Mexico and subsequently rapidly spread globally [1]. Two months after its emergence, the World Health Organization declared that this virus caused the first influenza pandemic of the 21st century [2]. Compared to those of seasonal influenza, the H1N1 virus demonstrated higher pathogenicity and increased infectivity in young healthy populations, leading to severe infections and complications [3]. Despite containment efforts, A(H1N1)pdm09 remains a persistent public health concern due to its ongoing circulation and potential viral mutations [4,5].

Infection with the H1N1 virus can cause various flu-like symptoms, such as fever, cough, sore throat, body aches, headache, chills, and fatigue [6]. In severe cases, patients may develop respiratory complications such as pneumonia and acute respiratory distress syndrome (ARDS), as well as systemic extrapulmonary manifestations [7]. H1N1 influenza infection may affect multiple organ systems, including the central nervous, cardiovascular, renal, musculoskeletal, hepatic, and endocrine systems, as well as ocular involvement and pregnancy-related complications [8]. There have been reports of liver dysfunction caused by H1N1 influenza infection. A clinical case report documented liver cell damage in a kidney transplant recipient infected with the H1N1 influenza virus [9]. Clinical reports indicated that infants infected with the H1N1 virus can experience impaired liver function and even liver failure [10,11]. Another clinical report showed severe H1N1 influenza infection and liver involvement in two previously healthy patients [12]. A clinical observational report revealed that 131 out of 224 H1N1 influenza-infected patients had abnormal liver function [13]. Although clinical evidence suggests that H1N1 may affect liver function, the mechanisms underlying H1N1 infection-induced liver dysfunction have rarely been reported.

In this study, an H1N1-infected BALB/c mouse model was utilized to investigate the impact of H1N1 infection on liver function and explore the underlying mechanisms involved. Our findings revealed significant liver pathology changes and elevated levels of serum aspartate transaminase (AST) and alanine transaminase (ALT) compared to those in control mice, indicating liver pathological damage and functional injury in H1N1-infected mice. mRNA-seq analysis revealed significant alterations in multiple genes affecting liver function after H1N1 infection, some of which mediate inflammatory responses involving monocyte adhesion/activation, and neutrophil/macrophage infiltration. Multiplex cytokine analysis identified marked inflammatory responses across tissues after infection: lung tissues showed increased levels of G-CSF, eotaxin, INF-gamma, IL-6, IP-10, KC, MCP-1, MIP-1alpha, and TNF-alpha; serum analysis revealed significant elevations in G-CSF, eotaxin, INF-gamma, IL-6, IL-10, IP-10, and MIP-1alpha; while liver tissues demonstrated particularly pronounced increases in eotaxin, KC, and IP-10. Hematological analysis revealed significant increases in monocyte, leukocyte, and neutrophil counts after H1N1 infection, suggesting that a systemic inflammatory response was triggered by H1N1. Collectively, these results suggest that H1N1 infection triggers a comprehensive inflammatory cascade that likely contributes to the observed liver damage. Our findings provide insights into the potential mechanisms of H1N1-induced liver injury, which may inform therapeutic strategies for human cases.

## 2. Materials and Methods

### 2.1. Virus

The H1N1 influenza virus strain (A/Michigan/45/2015) was kindly provided by Professor Guoyang Liao. The virus was propagated in specific pathogen-free (SPF) chicken embryos at a dilution of 1:105 in phosphate-buffered saline (PBS, pH 7.8–8.0). The harvested allantoic fluid was then tested, revealing a hemagglutination titer of 1:1024, and the virus titer, as measured by the plaque assay, was 1 × 10^7^ plaque-forming units (PFU)/mL. Prior to use, the virus-containing allantoic fluid was diluted to the desired concentration using sterile PBS.

### 2.2. Animal Experiments and the Ethics Statement

The 6–8-week-old female BALB/c mice were obtained from the Department of Laboratory Animals, Institute of Medical Biology, Chinese Academy of Medical Sciences (License No. SCXK (Dian) 2022-0002). The mice were housed in individually ventilated cages within a biosafety level 2 facility (License No. SYXK(Dian) K2022-0006, Registration No. 2022SW0013) under controlled environmental conditions (22–25 °C, 40–60% relative humidity, 12 h light–dark cycle). All animal experiments were approved by the Experimental Animal Ethics Committee of the Institute of Medical Biology, Chinese Academy of Medical Sciences(Approval ID: DWSP202307003), and all procedures were conducted in accordance with the protocols approved by the Ethics Committee.

The mice were randomly divided into two groups: the PBS group and the H1N1 group. H1N1-infected mice were intranasally inoculated with 50 μL of a 1 × 10^5^ PFU virus, while the PBS group received the same volume of PBS using the same method. Nasal and throat swabs were collected for viral load analysis. At 3 and 5 days post-infection (dpi), the mice were euthanized under isoflurane anesthesia (induction: 3–4% isoflurane at 300–400 mL/min airflow). Lung, turbinalia and liver tissues were collected on 3 and 5 dpi for viral load quantification. Lung and liver tissues on 5 dpi were collected for histopathological and transcriptomic (mRNA-seq) analyses. Whole blood samples on 5 dpi were obtained for hematological examination. Serum was collected and stored at −80 °C for further studies.

### 2.3. Detection of Serum ALT and AST Levels

ALT and AST levels in the serum were quantified using the Alanine Aminotransferase Assay Kit (Nanjing jiancheng, Nanjing, China) and the Aspartate Aminotransferase Assay Kit (Nanjing jiancheng, China), respectively. Assays were performed according to the manufacturer’s instructions.

### 2.4. Quantitative Polymerase Chain Reaction (qPCR)

RNA extraction was carried out using the zol™ RNA MiniPrep Plus kit (R2052; Zymo, Irvine, CA, USA) according to the manufacturer’s instructions. After tissue weighing, 800 μL trizol was added, followed by homogenization. Then, 200 μL supernatant was used for RNA extraction. After collecting nasal and throat swabs, add 800 μL of trizol and vortex thoroughly then extract 200 ul of RNA. The viral load was determined using the TaqMan Fast Virus 1-Step Master Mix (Thermo Fisher Scientific, Waltham, MA, USA) on the CFX384 Touch Real-Time PCR Detection System (Bio-Rad, Hercules, CA, USA). The viral load of tissues was expressed as viral copies per gram tissues. The viral load of nasal and throat swabs was expressed as viral copies per milliliter of trizol. The RTqPCR conditions consisted of an initial step at 25 °C for 2 min, followed by 15 min at 50 °C, and 2 min at 95 °C. This was followed by 40 cycles of 5 s at 95 °C and 31 s at 60 °C. The gene-specific primers and probes for the H1N1 virus were as follows: F: AAGACAAGACCAATYYTGTCACCTCT; R: TCTACGYTGCAGTCCYCGCT; P: FAMTYACGCTCACCGTGCCCAGTGTAMRA. RT-qPCR was employed to quantitatively analyze the mRNA expression levels of key genes in tissues. The RNA was reverse transcribed into cDNA using the NovoScript Plus All-in-one 1st Strand cDNA Synthesis SuperMix kit (Novoprotein, Suzhou, China). Quantitative PCR (qPCR) was performed on the CFX384 Touch Real-Time PCR Detection System (Bio-Rad) using the NovoStart SYBR qPCR SuperMix Plus kit (Novoprotein, Suzhou, China). The RT-PCR conditions included an initial denaturation step at 95 °C for 1 min, followed by 40 cycles of amplification at 95 °C for 20 s and 60 °C for 1 min. The mRNA expression levels of the target genes were normalized using GAPDH. The names and sequences of the gene-specific primers are presented in Supplementary Table S1.

### 2.5. Histopathological Evaluation

Fixation of fresh tissue samples was performed using 10% neutral buffered formalin. The tissues were then embedded in paraffin and sliced into 5 μm sections, followed by staining with hematoxylin and eosin (H&E). The slides were scanned using a 3DHISTECH scanner and evaluated by experienced pathologists using the CaseViewer software 2.4.0 provided by the manufacturer (3DHISTECH, Budapest, Hungary). The scoring method for pathological assessment was based on the reference [14]. The standardized criteria for histopathological evaluation have been provided in Supplementary Table S2.

### 2.6. Detection of Inflammatory Factors

Lung and liver tissues were quantitatively homogenized in 300 μL PBS at low temperature. Milliplex Mouse Cytokine/Chemokine Magnetic Bead Panel (Millipore, Burlington, MA, USA) was performed on a Bio-Plex 200 system (Bio-Rad, USA) to analyze the lung, liver, and serum samples, following the manufacturer’s instructions. The panel of inflammatory cytokines included Eotaxin, G-CSF, GM-CSF, IFN-G, IL-1a, IL-1b, IL-6, IL-7, IL-10, IL-15, IL-17, IP-10, MCP-1, MIP-1a, VEGF, and TNF-a.

### 2.7. Hematological Examination

The fresh blood samples on 5 dpi were collected in EDTA-coated tubes via retro-orbital bleeding at designated time points. Complete blood count (CBC) was performed using an automated hematology analyzer (Mindray, Shenzhen, China) according to the manufacturer’s protocol, measuring: monocyte, White blood cell, Neutrophil and its %, Llymphocyte%, Eosinophill % and so on.

### 2.8. mRNA Seq

Tissues were inactivated in 800 ul trizol and transported on dry ice to Shanghai Bohao Biological Co., Ltd. (Shanghai, China) for sequencing. The brief method was as follows: Total RNA was extracted from liver or lung; libraries were prepared according to Illumina standard instruction (VAHTS Universal V6 RNA-seq Library Prep Kit forIllumina^®^); evaluate the concentration and size distribution of cDNA libraries using the Agilent 4200 biological analyzer; sequencing with Illumina novaseq6000. The protocol of high-throughput sequencing strictly follows the manufacturer’s instructions (Illumina). Significantly differentially expressed genes (DEGs) were defined as genes with a false detection rate (FDR) value lower than the threshold (Q < 0.05) and fold-change > 2 using edgeR 3.2.0 software (Walter and Eliza Hall Institute of Medical Research, Melbourne, Australia).

### 2.9. Statistical Analysis

Statistical analysis was performed with GraphPad Prism software (9.4.0). All data are expressed using mean ± SEM. Unpaired 2-tailed *t*-test was used to compare differences between two groups. Statistical significance was defined as *p* < 0.05. The correlation analyses among clinical parameters, as well as between pulmonary/hepatic/serum inflammatory factors and clinical indicators, were visualized using correlation heatmaps generated by ChiPlot (https://www.chiplot.online/) (accessed on 7 August 2025).

## 3. Results

### 3.1. H1N1-Infected BALB/c Mice Developed Severe Pneumonia

**Figure 1:**
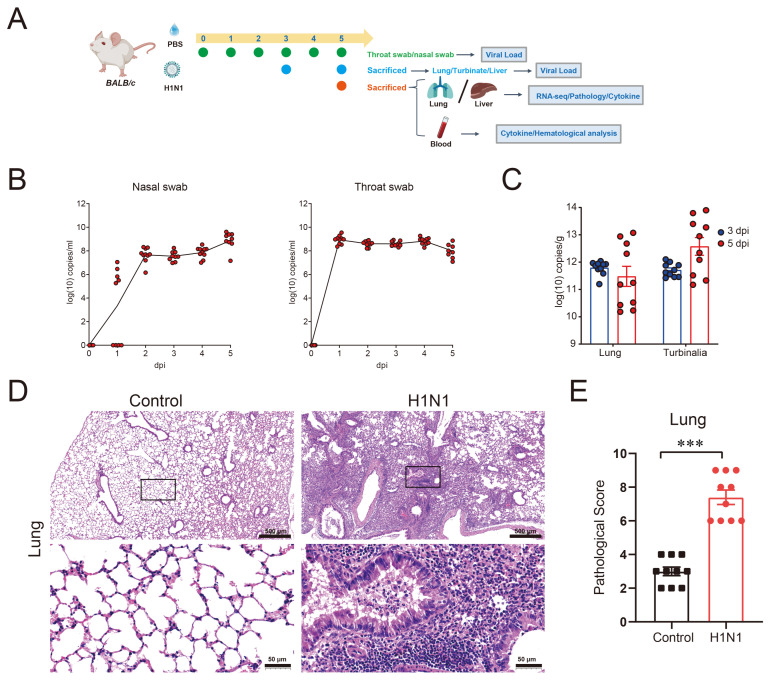
H1N1 infection caused severe pneumonia in BALB/c mice: (**A**) experimental design timeline; (**B**) viral load in throat swabs and nasal swabs from H1N1-infected and control mice; (**C**) viral loads in lung and turbinalia on 3 dpi and 5 dpi; (**D**) representative H&E stain of lungs on 5 dpi. Red represents cytoplasm, purple represents nuclei; (**E**) histopathological scores of lung tissues on 5 dpi. The differences between the two groups were determined using unpaired *t*-test. *** *p* < 0.001. Each group contains 9–10 mice.

H1N1 infection is primarily transmitted via the respiratory route, making viral replication in the respiratory tract a key indicator of disease severity. In this study, mice were intranasally inoculated with H1N1. Viral load dynamics were monitored through daily collection of throat and nasal swabs post-infection. On 3 and 5 dpi, the mice were sacrificed to measure viral loads in the lungs and turbinalia (Figure 1A). The results demonstrated a sharp increase in the viral load in nasal swabs by 2 dpi, which remained consistently high thereafter (Figure 1B). Similarly, throat swabs showed a significant rise in viral levels as early as 1 dpi, maintaining high load throughout the observation period (Figure 1B). Notably, both the turbinalia and lungs exhibited substantial viral replication on 3 and 5 dpi (Figure 1C). More importantly, histological examination of H&E-stained lung sections on 5 dpi showed prominent lymphocyte infiltration, bronchial epithelial cell adhesion, bronchial obstruction, and thrombus formation in the blood vessels, indicating robust pulmonary inflammation and potential hypoxia (Figure 1D). Pathological scoring revealed significantly more severe lung damage in infected mice compared to controls. The lung tissue exhibited a significantly greater pathological score than controls (Figure 1E). Collectively, these findings demonstrated that H1N1 infection induced severe pneumonia in BALB/c mice.

### 3.2. H1N1 Infection Induces Liver Injury and Elevates Serum ALT/AST Levels in BALB/c Mice

**Figure 2:**
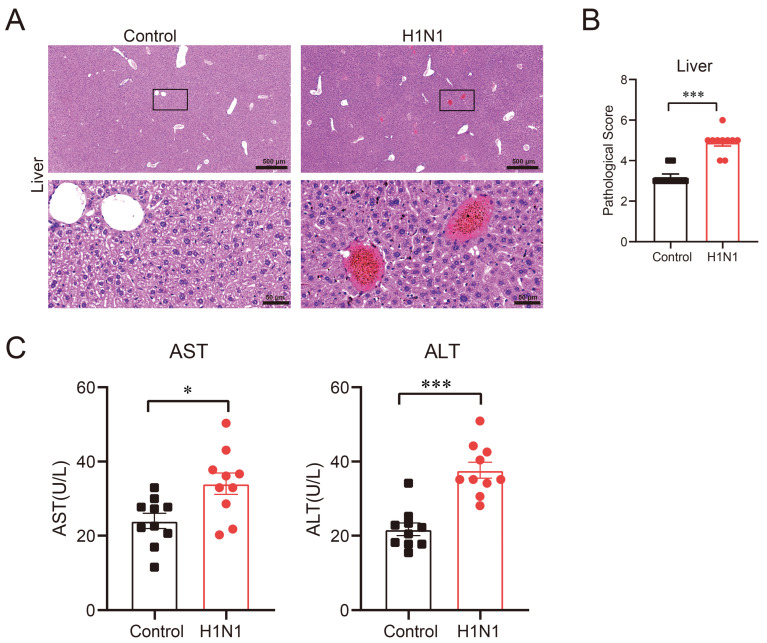
H1N1 infection induced liver injury in BALB/c mice. Histopathological examination of liver tissues and serum ALT/AST levels were measured on 5 dpi: (**A**) representative H&E-stained liver sections on 5 dpi. Red represents cytoplasm, purple represents nuclei; (**B**) histopathological scores of liver tissues on 5 dpi; (**C**) serum ALT and AST levels on 5 dpi. Each group contains 10 mice. Data are presented as mean ± SEM (*n* = 10 mice/group). Statistical significance was determined by unpaired Student’s *t*-test (* *p* < 0.05, *** *p* < 0.001).

Subsequently, we examined the effects of H1N1 influenza virus infection on liver pathology in mice. Although qPCR analysis failed to detect viral RNA in liver tissue, histopathological and biochemical evidence indicated significant liver damage. By day 5 post-infection, H1N1-infected mice exhibited prominent lymphocyte infiltration, vascular thrombosis, and marked hemorrhage in liver tissue (Figure 2A). Histopathological examination demonstrated significantly higher liver pathology scores in H1N1-infected mice compared to controls (Figure 2B). As elevated ALT and AST levels are established markers of hepatocellular injury—with ALT indicating acute damage (e.g., viral hepatitis or drug-induced necrosis) and AST reflecting severe necrosis [15,16]—we measured serum enzyme levels. Serum ALT and AST levels were significantly elevated in H1N1-infected mice versus controls (Figure 2C). These findings demonstrated that H1N1 infection impaired liver structure and function in mice, even in the absence of detectable hepatic viral RNA.

### 3.3. mRNA-Seq Analysis Reveals Abnormal Liver Function in H1N1-Infected BALB/c Mice

**Figure 3:**
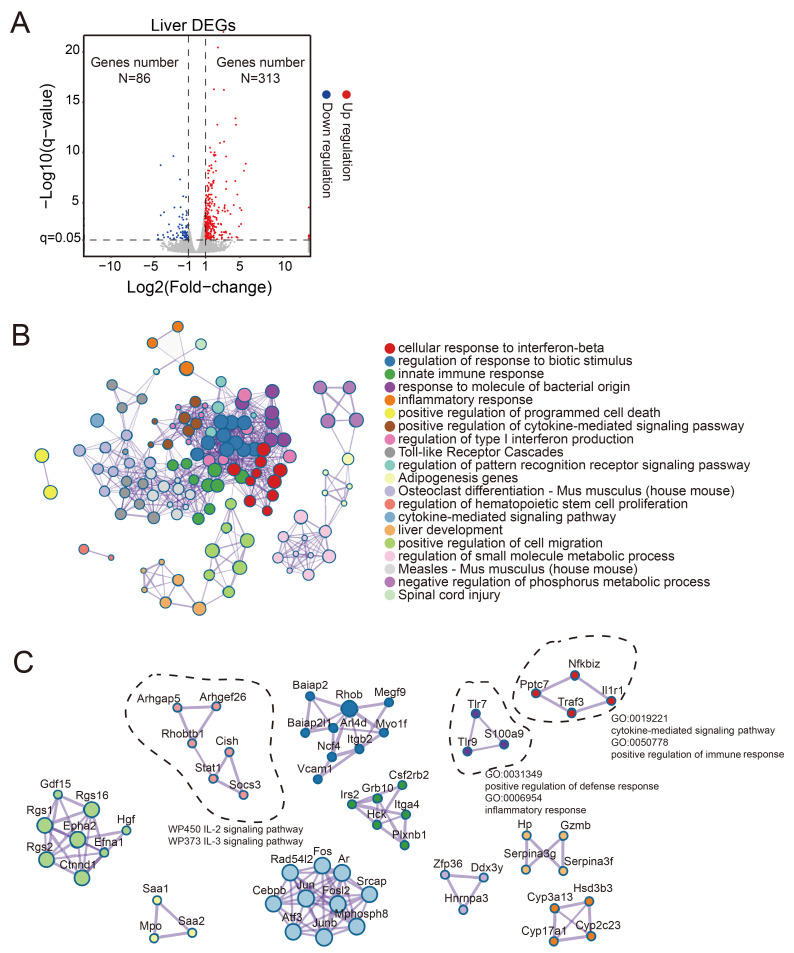
Transcriptomic profiling revealed H1N1-induced liver dysfunction in BALB/c mice: (**A**) volcano plot of DEGs in liver tissues between H1N1-infected and control groups on 5 dpi. Blue dots represent downregulated genes (*n* = 86), red dots represent upregulated genes (*n* = 313) (|log2FC| > 1, FDR < 0.05); (**B**) GO enrichment analysis of DEGs. Bubble size reflects the number of genes in each pathway; (**C**) protein–protein interaction network of GO-enriched terms. Node size corresponds to the number of interacting genes.

To evaluate H1N1-induced transcriptional changes in the liver, we collected tissue samples from H1N1-infected or mock-infected mice on 5 dpi, and performed mRNA-seq analysis. Compared to the control group, H1N1-infected mice exhibited significant alterations in hepatic gene expression, with 313 upregulated and 86 downregulated genes (Figure 3A). Gene Ontology (GO) analysis of the differentially expressed genes (DEGs) in the liver revealed enrichment in pathways associated with the type I interferon response to viruses, inflammatory response, liver development, small molecule metabolism, and lipid metabolism (Figure 3B). The protein–protein interaction network further indicated that these DEGs were mainly involved in the cytokine activity and the positive regulation of immune defense responses (Figure 3C). Collectively, these findings demonstrated that H1N1 infection disrupted hepatic gene expression, particularly in pathways governing liver function and inflammatory regulation.

### 3.4. Hepatic Inflammatory Gene Signatures Induced by H1N1 Infection

**Figure 4:**
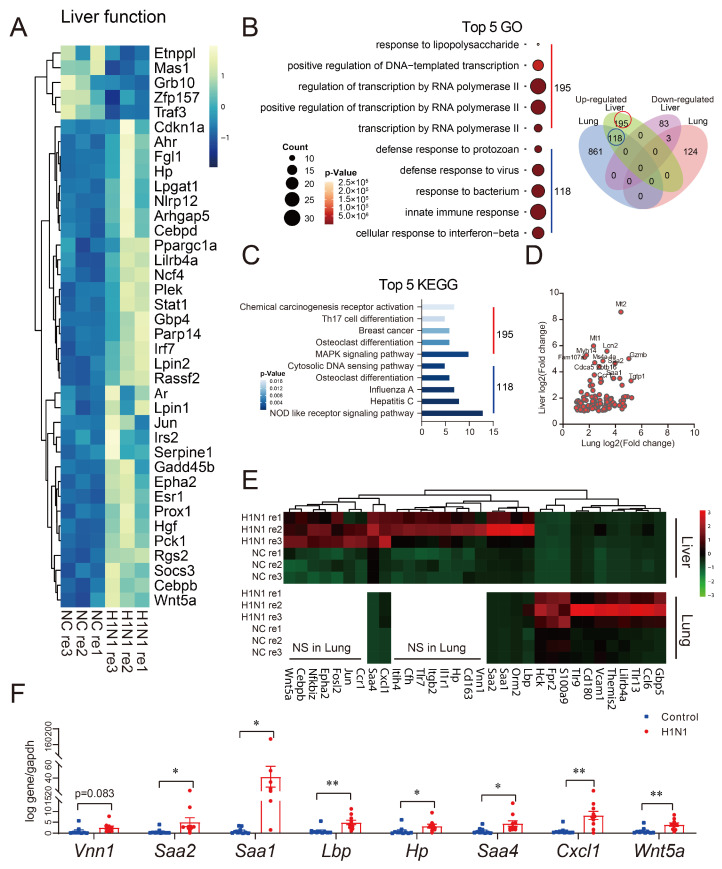
Comparative transcriptomic analysis of inflammatory responses in liver and lung tissues following H1N1 infection. Liver and lung tissues from H1N1-infected or mock-infected mice on 5 dpi for mRNA-seq analysis: (**A**) heatmap of liver function-related DEGs; (**B**) GO analysis of shared liver–lung DEGs and liver-specific DEGs (vs. lung), respectively; Venn diagram of DEGs in the lung and liver after H1N1 infection; (**C**) KEGG pathway enrichment analysis of shared liver–lung DEGs and liver-specific DEGs (vs. lung), respectively; (**D**) scatter plot of DEGs in lung and liver after H1N1 infection; (**E**) heatmaps of DEGs related to inflammatory response in the lung and liver tissues; (**F**) RT-qPCR validation of 12 key inflammation-related DEGs (* *p* < 0.05, ** *p* < 0.01).

Transcriptomic analysis revealed that H1N1 infection induced significant alterations in hepatic gene expression profiles, with DEGs being predominantly enriched in liver function-related pathways, including liver development, small molecule metabolic processes, and lipid metabolism. A heatmap of the DEGs associated with these pathways is shown in Figure 4A. The DEGs *Ahr*, *Cebpb*, *Hgf*, *Hp*, *Jun*, *Pck1*, *Prox1*, *Fgl1*, *Ar*, *Arhgap5*, *Epha2*, *Esr1*, *Wnt5a*, and *Irs2* were related to liver development. The upregulation of *Ahr*, *Cdkn1a*, *Socs3*, *Lpin1*, *Lilrb4a*, *Hgf*, *Jun*, *Gbp4*, *Gadd45b*, *Ppargc1a*, *Rgs2*, *Plek*, *Rassf2*, *Nlrp12*, *Irs2*, and *Parp14,* along with the downregulation of *Mas1* and *Grb10*, showed significant enrichment in pathways associated with phosphorus metabolism regulation. The observed changes in the adipocytokine signaling pathway and glycerophospholipid metabolism following H1N1 infection may be attributed to the effects of DEGs, including *Lpin1*, *Pck1*, *Lpin2*, *Lpgat1*, *Ahr*, *Cebpb*, *Cebpd*, *Socs3*, *Gadd45b*, *Serpine1*, *Ppargc1a*, *Stat1* and *Irs2* upregulation and *Etnppl* downregulation (Figure 4A). The intersection of DEGs in the liver and lung after infection indicated that the common DEGs were mainly upregulated, with 118 genes upregulated upon infection in both the lungs and liver, while 195 genes were upregulated only in the liver (Figure 4B). DEGs in both liver and lung tissues were predominantly enriched in pathways related to the cellular response to interferon-beta, response to viruses, innate immune response, and inflammatory response. Notably, multiple DEGs in the liver are related to MAPK signaling (Figure 4B,C). The expression of several DEGs, such as *Mt1*, *Gzmb*, *Tgtp1*, and *Lcn2*, was significantly upregulated in both the liver and lung (Figure 4D). Further analysis of the DEGs involved in the inflammatory response in the liver and lung revealed distinct patterns between the liver and lung. Genes such as *Gbp5*, *Ccl6*, *Tlr13*, *Lilrb4a*, *Themis2*, *Vcam1*, *Cd180*, *Tlr9*, *S100a9*, *Fpr2*, and *Hck* showed significantly higher expression in the lung compared to the liver. In contrast, *Lbp*, *Orm2*, the serum amyloid A (SAA) protein family, and *Cxcl1* were more strongly upregulated in the liver. Additionally, *Vnn1*, *Cd163*, *Hp*, *Il1r1*, *Itgb2*, *Tlr7*, *Cfh*, *Itih4*, *Cxcl1*, *Saa4*, *Ccr1*, *Jun*, *Fosl2*, *Epha2*, *Nfkbiz*, *Cebpb*, and *Wnt5a* exhibited differential expression exclusively in the liver (Figure 4E). Among these genes, *Ncf4*, *Lbp*, *Fos*, *Jun*, *Hspa2*, and *Vcam1* were involved not only in monocyte adhesion and activation but also in neutrophil and macrophage infiltration. qPCR validation revealed significant upregulation of *Vnn1*, *LBP*, *Hp*, *Cxcl-1*, *Wnt5a*, *Epha2*, and particularly the SAA protein family, which was closely associated with the inflammatory response, in the liver (Figure 4F).

### 3.5. H1N1 Infection Triggers Systemic Inflammatory Responses in Mice

**Figure 5:**
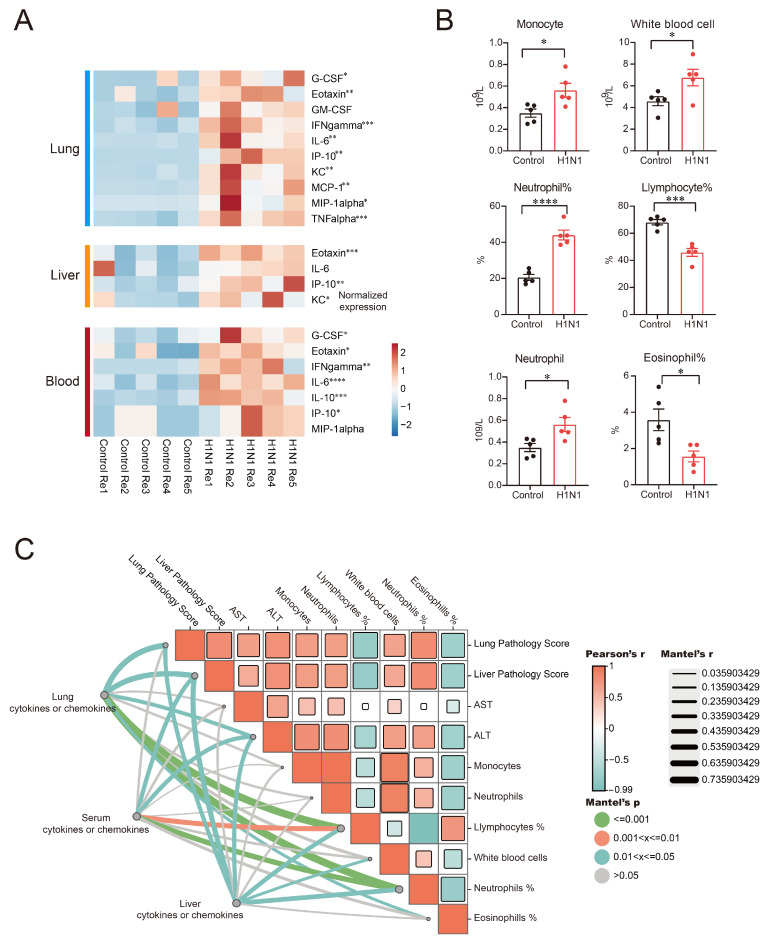
Systemic inflammatory profiling and hematological alterations induced by H1N1 infection: (**A**) heat maps of inflammatory factors in the lungs (pg/g tissue), liver (pg/g tissue), and serum (pg/mL) on 5 dpi. * *p* < 0.05, ** *p* < 0.01, *** *p* < 0.001, **** *p* < 0.0001 by unpaired Student’s *t*-test; (**B**) hematological analysis of infected and uninfected mice on 5 dpi. Data presented as mean ± SEM (*n* = 5/group). * *p* < 0.05, *** *p* < 0.001, **** *p* < 0.0001 by unpaired Student’s *t*-test; (**C**) statistical correlation analyses. (**Right**) Pearson correlation matrix of clinical parameters; (**Left**) Mantel test results assessing associations between clinical indicators and (i) lung, (ii) liver, and (iii) serum cytokines/chemokines.

No H1N1 virus was detected in the liver, suggesting that hepatic injury in infected mice may not result from direct viral cytopathic effects. Transcriptomic analysis revealed a series of inflammatory responses in the liver and lung tissues of H1N1-infected mice. We then examined the changes in multiple inflammatory factors across different compartments on day 5 post-H1N1 infection in mice. We found that H1N1-infected mice showed significantly elevated levels of G-CSF, eotaxin, GM-CSF, IFN-gamma, IL-6, IP-10, KC, MCP-1, MIP-1alpha, and TNF-alpha in lungs of the H1N1-infected group compared to control (Figure 5A). Serum analysis showed significant increases in G-CSF, eotaxin, IFN-gamma, IL-6, IL-10 and IP-10 (Figure 5A). In liver tissue, eotaxin, KC, and IP-10 were significantly elevated, with IL-6 displaying a non-significant upward trend (Figure 5A). The monocyte count, white blood cell count, neutrophil count, and neutrophil percentage were significantly increased in the hematological analysis of whole blood from infected mice, while the lymphocyte count and eosinophil percentage were significantly decreased (Figure 5B). Additionally, we conducted Pearson correlation analysis among clinical parameters (including histopathological scores of lung and liver tissues, liver function markers [AST, ALT], and complete blood count) from both H1N1-infected and uninfected mice. Subsequently, Mantel tests were performed to examine the associations between: (i) lung, (ii) liver, and (iii) serum inflammatory factors with these clinical indicators. Notably, these analyses demonstrated significant positive correlations between inflammatory factors across all three compartments (lung, liver, and serum) and both organ pathology scores, as well as with ALT levels (Figure 5C). Collectively, these findings suggest that H1N1-triggered systemic inflammation in mice appears associated with liver damage.

## 4. Discussion

Liver inflammation and injury were consequences of the liver’s immune response to hepatotropic viruses such as hepatitis A, B, C, and E viruses [17]. Additionally, non-hepatotropic viruses, such as respiratory viruses (e.g., SARS-CoV-2 and influenza viruses), herpes viruses, parvovirus, and adenovirus, can potentially invade the liver [18,19]. These infections of the liver may cause a spectrum of liver dysfunction, ranging from mild biochemical abnormalities to fulminant liver failure [18]. Here, H1N1 infection in BALB/c mice induced marked liver injury (elevated ALT/AST) and histopathological changes, establishing this model for studying respiratory virus-mediated liver dysfunction.

Our observations demonstrated that H1N1 viral replication in infected mice occurred primarily in the respiratory system, with no detectable virus RNA in the liver. This suggested that H1N1-induced liver injury may not result from direct viral activity in liver tissue. Previous studies have reported similar “incidental injury” in the livers of C57BL/6 (B6) mice following respiratory A/Kawasaki/86 (H1N1) virus infection, despite the absence of viral replication [20]. While Zhang et al. reported detectable influenza virus (A/WSN/33) in mouse liver using both RT-qPCR and immunohistochemistry following lethal-dose infection (LD50) [21], our negative results may reflect strain-specific differences in H1N1 viral kinetics affecting viral clearance rates; the distinct pathological progression induced by our sublethal challenge dose compared to their lethal dose protocols. A Japanese case report further identified hypoxic hepatitis as a critical contributor to influenza-associated acute liver failure [22]. Consistent with this mechanism, our experimental data demonstrated bronchial obstruction through histological analysis, suggesting systemic hypoxia may represent a plausible pathway for H1N1-induced hepatic damage.

Cytokines serve as crucial mediators in both host defense and homeostasis maintenance [23]. The liver, being particularly sensitive to systemic imbalances, responds to such perturbations through multiple regulatory mechanisms. Pearson correlation analysis in H1N1-infected mice revealed a positive correlation between the lung pathology severity, hepatic inflammatory damage, and liver biochemical markers (serum AST/ALT). Mantel analysis further demonstrated that post-infection inflammatory cytokine levels in the lungs, blood, and liver were positively correlated with histopathological damage in both the liver and lung tissues. Interestingly, inflammatory cytokines in the lungs, blood, and liver showed a strong positive correlation with ALT; their association with AST was nonsignificant (Figure 5C)—potentially reflecting ALT’s superior sensitivity for detecting hepatic injury [24,25]. The correlation between elevated ALT/AST levels and systemic inflammation induced by H1N1 infection requires further mechanistic investigation. During viral infection, pro-inflammatory cytokines (such as IL-6, TNF-α, and IFN-γ) activate and recruit immune cells (including neutrophils and monocytes) to the site of infection, mediating apoptosis and clearance of virus-infected cells [26]. These cytokines enter systemic circulation and not only induce changes in peripheral immune cell profiles (e.g., increased neutrophils and decreased lymphocytes), but can also affect the function of distal organs through the circulatory system [27]. H1N1 infection in humans clinically manifests as severe pneumonia, which can progress to a cytokine storm and ultimately result in multiple organ failure [28,29]. Our study demonstrates that even sub-lethal doses of H1N1 virus infection in mice can lead to hepatic dysfunction (e.g., elevated ALT levels) and histopathological alterations in liver tissue (including congestion, zonal necrosis, and macrophage infiltration). These findings suggest that in the clinical management of H1N1 infection, in addition to antiviral therapy and respiratory support, close monitoring of liver function (particularly ALT levels) and timely intervention are warranted to mitigate the risk of multi-organ failure.

**Figure 6:**
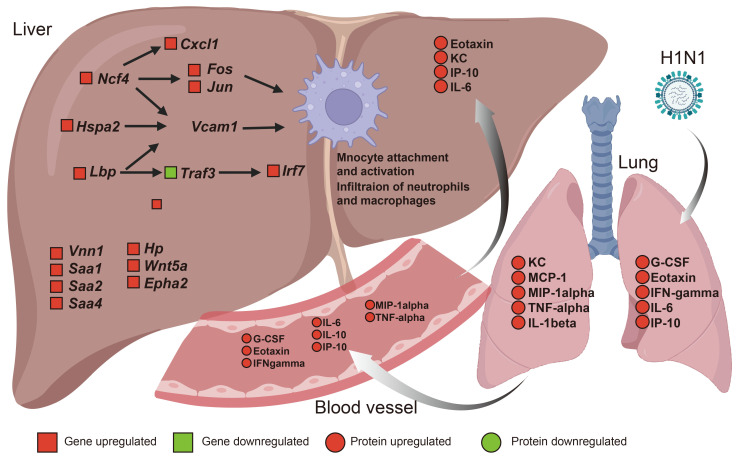
Working model diagram of liver injury caused by H1N1 infection. H1N1 infection induces systemic inflammation, marked by elevated inflammatory mediators in the lungs, serum, and liver. Although no direct viral presence is detected in hepatic tissue, the observed liver injury—evidenced by increased AST/ALT levels and histopathological alterations—likely results from inflammatory cascades involving monocyte–endothelial adhesion, neutrophil/macrophage infiltration, and dysregulated gene expression pathways.

Following H1N1 infection, we observed significant upregulation of Ncf4 (p40phox) in the liver. Ncf4, known as p40phox, is a cytoplasmic protein that binds to NCF1 and NCF2 to form the membrane NOX2 complex to induce reactive oxygen species (ROS) reactions [30]. This elevated Ncf4 expression may exacerbate hepatic oxidative stress through enhanced ROS production. CXCL1 is a potent neutrophil chemotactic factor. Studies have reported that elevated CXCL1 expression positively correlates with neutrophil infiltration in liver samples from drug-induced liver failure [31]. We observed significant upregulation of CXCL1 transcriptional levels in hepatic tissue, with transcriptomic analysis further revealing functional interactions between NCF4 and CXCL1. Histopathological examination confirmed inflammatory cell infiltration in the liver, suggesting that both NCF4 and CXCL1 may collectively contribute to the recruitment of inflammatory cells during H1N1-induced hepatic pathology (Figure 6). The interaction of Ncf4 with the downstream molecules Fos, Jun, and Vcam1 was closely associated with monocyte adhesion/activation, as well as lymphocyte and macrophage infiltration (Figure 6). Lipopolysaccharide (LPS)-binding protein (LBP), which was primarily produced in liver cells, acts as a secretory type I acute-phase protein and plays a crucial role in innate immune responses [32]. Initially, LPS forms a complex with LBP, which was followed by binding to CD14 and the MD-4/MD-2 complex [32,33], ultimately activating signaling pathways and inducing the production of cytokines and other proinflammatory mediators [32]. Changes in the expression of the *Lbp*, *Traf3*, and *Irl7* genes may be implicated in hepatic inflammatory responses (Figure 6).

Transcriptomic analysis revealed significant upregulation of serum SAA family genes in the liver on 5 dpi. The SAA protein family consists of multiple isoforms, with SAA1 and SAA2 serving as the major acute-phase reactants alongside C-reactive protein (CRP) [34]. During this phase, the serum SAA concentration rapidly increases in response to trauma, infection, or other stimuli as an early immune response to viral infection. SAA is synthesized primarily in the liver, and SAA fragments can form pathological amyloid fibrils characteristic of secondary amyloidosis [35]. These fibrils may disrupt normal physiological functions and lead to organ failure. During systemic inflammation, increased levels of proinflammatory cytokines such as IL-6, IL-1β, and TNF drive SAA overexpression [36,37]. Prolonged high levels of SAA can promote a series of pathological events, including protein misfolding, fragmentation, and aggregation into highly ordered amyloid fibrils [38]. The increase in SAA levels is closely related to platelet aggregation [39], neutrophil activation [40], and immune cell migration [41]. Studies demonstrate that SAA can significantly promote the expression and secretion of the chemokine CCL20 in monocytes by activating the MAPK signaling pathway [42], while also enhancing the inflammatory response of macrophages through MAPK signaling activation [43]. Our KEGG pathway analysis results reveal that in H1N1-infected mouse models, the MAPK signaling pathway shows significant activation levels in liver tissues (Figure 4C). Whether H1N1-induced liver injury involves SAA-triggered MAPK pathway activation remains an interesting question for future studies. In summary, elevated hepatic SAA transcript levels play a significant role in both systemic inflammation and liver injury induced by H1N1 infection (Figure 6). Emerging evidence suggests that systemic inflammation may contribute to liver injury by disrupting the gut microbiota and impairing the gut–liver axis [44]. H1N1 infection may induce systemic inflammation and hepatic injury through multiple mechanisms, including infection-induced microbiota dysbiosis, which requires more comprehensive and systematic investigation. Additionally, our findings, based on biological sample analyses from the early post-infection phase, highlight the need for future studies with larger sample sizes and longer follow-up after H1N1 infection. Whether H1N1 leads to persistent liver damage remains uncertain, and if it does, the underlying mechanisms may differ from those involved in the acute phase of infection.

## 5. Conclusions

Our study demonstrated that H1N1 infection leads to severe pathological damage in the liver, characterized by marked lymphocyte infiltration, vascular thrombosis, and significant hemorrhage in liver tissues. Additionally, there were significant increases in ALT and AST levels. Although viral RNA was undetectable in hepatic tissue, H1N1 infection triggered a systemic inflammatory response that initiated coordinated gene regulatory cascades in the liver, resulting in hepatic inflammation, monocyte activation, and robust infiltration of neutrophils and macrophages. The transcriptomic profiling data provide compelling molecular evidence supporting these pathological manifestations, collectively advancing our understanding of the intricate mechanisms underlying H1N1-induced hepatotoxicity.

## Data Availability

All research data were included in the article. The original data for RNA sequencing in this study can be obtained on NCBI SRA with registration number PRJNA1048544.

## Supplementary Materials

The following supporting information can be downloaded at: https://www.mdpi.com/article/10.3390/v17081132/s1, Supplementary Table S1: rimer sequences for RT-qPCR; Supplementary Table S2: Pathological scoring criteria.
